# A practical approach for geographic prioritization and targeting of insecticide-treated net distribution campaigns during public health emergencies and in resource-limited settings

**DOI:** 10.1186/s12936-021-04028-y

**Published:** 2022-01-04

**Authors:** Alyssa J. Young, Will Eaton, Matt Worges, Honelgn Hiruy, Kolawole Maxwell, Bala Mohammed Audu, Madeleine Marasciulo, Charles Nelson, James Tibenderana, Tarekegn A. Abeku

**Affiliations:** 1 Tulane University School of Public Health and Tropical Medicine, New Orleans, LA USA; 2 Malaria Consortium, Abuja, Nigeria; 3 National Malaria Elimination Programme, Abuja, Nigeria; 4 Malaria Consortium, Raleigh, USA; 5 Malaria Consortium, London, UK

**Keywords:** *Plasmodium falciparum*, Malaria, Vector control, Prioritization scheme, Intervention targeting, Insecticide-treated nets, ITN distribution campaign, GIS-AHP, Analytic hierarchy process, INLA-SPDE, MCDA

## Abstract

**Background:**

The use of data in targeting malaria control efforts is essential for optimal use of resources. This work provides a practical mechanism for prioritizing geographic areas for insecticide-treated net (ITN) distribution campaigns in settings with limited resources.

**Methods:**

A GIS-based weighted approach was adopted to categorize and rank administrative units based on data that can be applied in various country contexts where *Plasmodium falciparum* transmission is reported. Malaria intervention and risk factors were used to rank local government areas (LGAs) in Nigeria for prioritization during mass ITN distribution campaigns. Each factor was assigned a unique weight that was obtained through application of the analytic hierarchy process (AHP). The weight was then multiplied by a value based on natural groupings inherent in the data, or the presence or absence of a given intervention. Risk scores for each factor were then summated to generate a composite unique risk score for each LGA. This risk score was translated into a prioritization map which ranks each LGA from low to high priority in terms of timing of ITN distributions.

**Results:**

A case study using data from Nigeria showed that a major component that influenced the prioritization scheme was ITN access. Sensitivity analysis results indicate that changes to the methodology used to quantify ITN access did not modify outputs substantially. Some 120 LGAs were categorized as ‘extremely high’ or ‘high’ priority when a spatially interpolated ITN access layer was used. When prioritization scores were calculated using DHS-reported state level ITN access, 108 (90.0%) of the 120 LGAs were also categorized as being extremely high or high priority. The geospatial heterogeneity found among input risk factors suggests that a range of variables and covariates should be considered when using data to inform ITN distributions.

**Conclusion:**

The authors provide a tool for prioritizing regions in terms of timing of ITN distributions. It serves as a base upon which a wider range of vector control interventions could be targeted. Its value added can be found in its potential for application in multiple country contexts, expediated timeframe for producing outputs, and its use of systematically collected malaria indicators in informing prioritization.

**Supplementary Information:**

The online version contains supplementary material available at 10.1186/s12936-021-04028-y.

## Background

The use of data in informing malaria control efforts is increasingly being adopted by National Malaria Control Programmes (NMCPs). Data used to inform malaria interventions have expanded to encompass a variety of data points and covariates that are no longer limited to reported malaria case counts [1–3]. Improvements in the specificity and resolution at which interventions are targeted may not only increase the impact of a particular intervention but also facilitate optimal use of limited resources. The use of new types of insecticide-treated nets (ITNs) and chemicals for indoor residual spraying (IRS) due to insecticide resistance has resulted in increased vector control costs, which would make prioritization necessary in situations of limited funding. Furthermore, prioritization is especially relevant during public health emergencies such as the coronavirus disease 2019 (COVID-19) pandemic as health services in malaria-endemic countries have had to re-allocate funding and resources towards COVID-19 containment efforts [4]. The COVID-19 pandemic has not only affected countries in sub-Saharan Africa directly by increasing strain on an already overburdened healthcare infrastructure, but also indirectly through cessation or delay in other disease control activities, including those essential in preventing and treating malaria cases [4].

The objective of this work was to develop a practical, data-driven mechanism for prioritizing geographical areas for mass distribution of ITNs amidst a public health emergency and in situations of limited resources. Whereas the use of routine surveillance and malaria intervention coverage data is typically recommended when targeting malaria interventions, this approach aimed to create a simple approach that could be easily interpreted and replicated without a need for in-depth statistical modelling and that can be applied in various country contexts where *Plasmodium falciparum* transmission is reported. Additionally, this work sought to create an expedited approach for targeting ITNs where the timeline to construct, map and apply outputs would have to occur within an accelerated timeframe. The intended use of outputs was to prioritize which local government areas (LGAs) should receive precedence during ITN distribution campaigns. Results from Nigeria as a case study are used to illustrate application of the approach especially during the COVID-19 pandemic and in a setting with limited availability of funding for universal coverage at national and sub-national levels. This manuscript presents a technical yet practical methodology that malaria control programmes can adapt to their needs, along with recommendations as to which indicators could be considered while planning vector control interventions such as ITN campaigns in the context of limited resources and public health emergencies.

## Methods

### Approach

A prioritization methodology was created that can be used for systematically selecting areas that need to be targeted in an appropriate sequence with ITN distributions, while taking into consideration malaria transmission risks and a range of other relevant factors. Whereas Nigeria was used as a case study, this manuscript presents a general approach that can be adopted by any country or region experiencing malaria transmission that has: (1) access to sub-national malaria intervention coverage data; (2) conducted a Demographic and Health Survey (DHS) or Malaria Indicator Survey (MIS) within the past 3 years; (3) access to open-source spatial layers featuring data pertaining to covariates such as *P. falciparum* environmental suitability indices, educational attainment and population density; and, (4) available conflict and/or disruptive event data (if relevant). These widely available data sources can be accessed via country-specific government documents, such as national malaria control (or elimination) strategic plans and DHS/MIS reports and databases, as well as open-source data platforms (such as the Malaria Atlas Project (MAP) [5], Climate Hazards Group InfraRed Precipitation with Station (CHIRPS) [6], and Humanitarian Data Exchange [7]. Using a combination of data derived from these elements, risk scores were calculated for each LGA within Nigeria to rank the risk of these units to inform prioritization for ITN distribution.

In this exercise*, P. falciparum*-specific factors were utilized in calculating final risk scores at the LGA level, along with other factors that have impacts on malaria prevention and control in Nigeria. Data inputs that were included in the model (Table 1) were selected due to: (1) their availability in multiple countries and transmission contexts; (2) their relevance in characterizing malaria risk; and, (3) their ability to help identify discrepancies in ITN access. The latter two were achieved through focus group discussions with malaria researchers and literature reviews [8–12].

**Table 1:** List of input factors and corresponding method of extraction, weight, classification, rank, and risk characterization

Factor	Method of extraction/application	Weight (obtained using AHP)	Classification	Rank value	Risk characterization
CHIRPS mean annual rainfall (mm) from May 2019 to April 2020 [6]	Climate Hazards Group InfraRed Precipitation with Station (CHIRPS) data was used to calculate mean monthly rainfall (from May 2019 to April 2020) by LGA	0.16	> 207 mm	4	Very high
			148–207 mm	3	High
			92–147 mm	2	Moderate
			≤ 91 mm	1	Low
*P. falciparum* temperature suitability index [5]	Extracted from raster layer created by Malaria Atlas Project (MAP)	0.09	≤ 0.453	1	Low
			0.454–0.570	2	Moderate
			0.571–0.680	3	High
			> 0.680	4	Very high
Percentage of children aged 6–59 months who tested positive for malaria by microscopy [26]	Obtained from state-Level adjusted percentages reported in 2018 Nigeria DHS	0.07	> 42%	5	Very high
			33–42%	4	High
			23–32%	3	Moderate
			13–22%	2	Low
			< 12%	1	Very low
Number of years since last mass ITN distribution	Calculated for each LGA using dates reported in NMCP documents	0.19	≥ 6 years	4	Very high
			5 years	3	High
			3 years	2	Moderate
			< 3 years	1	Low
PBO net distribution in 2019	Obtained from NMCP operational plans and intervention coverage documents	0.09	Not distributed	1	High
			Distributed	0	Low
Proportion of households with at least 1 ITN per 2 people [26] *	Modelled using INLA-SPDE method on 2018 DHS data. Values provided here are Nigeria specific and are aggregated to the LGA level	0.15	< 23%	4	Very high
			23–34%	3	High
			35–48%	2	Moderate
			> 48%	1	Low
SMC coverage in 2019	Obtained from NMCP operational plans and intervention coverage documents	0.08	Not implemented	1	High
			Implemented	0	Low
Built-up area presence index (proxy for urban/rural designation) [25]	Extracted and aggregated to respective administrative unit (LGA) using SMOD raster layers	0.09	≤ 0.0084	4	Very high
			0.051–0.0085	3	High
			0.76–0.05	2	Moderate
			> 0.76	1	Low
Percentage of *de jure* population comprising lowest wealth quintile [26]	Obtained from state-level percentages reported in 2018 Nigeria DHS	0.06	> 40.8%	4	Very high
			24.6–40.8%	3	High
			8–24.5%	2	Moderate
			≤ 8%	1	Low
Internally displaced populations (resulting from armed conflict) in 2020 [7]	Obtained from data sets provided by Humanitarian Data Exchange	0.03	Present	1	High
			Not present	0	Low

**Fig. 1:**
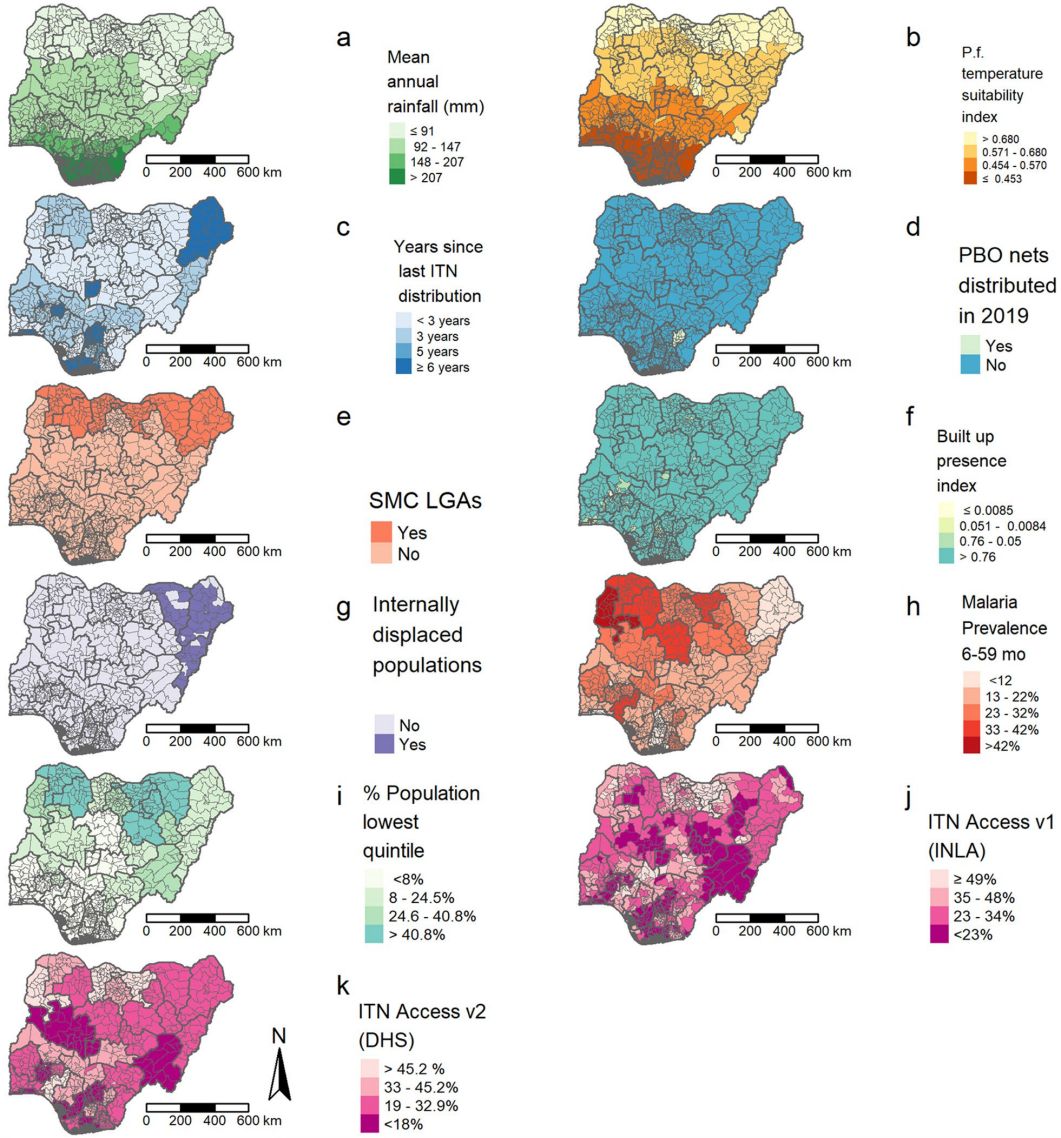
Geospatial risk classification of input factors (**a**–**j**) used to create final prioritization map, and alternative ITN access layer (**k**)

**Fig. 2:**
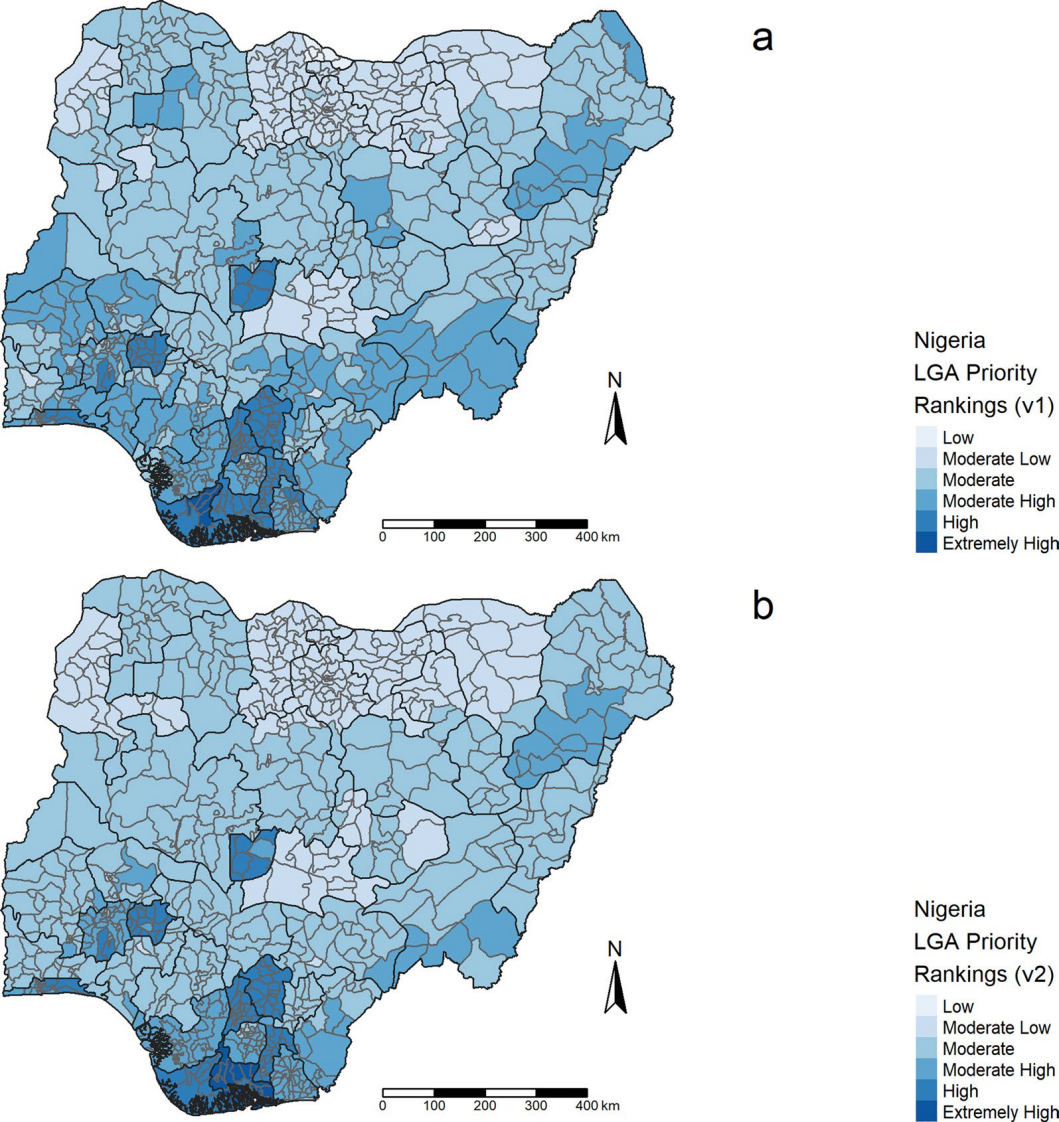
Final prioritization schemes using **a** the spatially interpolated ITN access layer *versus* **b** state level ITN access

*A sensitivity analysis was conducted using an alternative layer for ITN access (Fig. 1k). This layer featured state-level proportion of households with at least 1 ITN per 2 people and the following classification intervals: < 18% (very high); 18–32.9% (high); 33–45.2% (moderate); > 45.2% (low). Differences in final prioritization scheme outputs are illustrated in Fig. 2

The basis of this methodology is derived from a strategy developed by Hanafi-Bojd et al., where a geographic information system (GIS)-based weighted arithmetic and multiplicative approach was used to categorize and rank administrative units based on malaria hazard and risk in the context of targeting interventions in a setting with *Plasmodium vivax* and *P. falciparum* transmission [8]. Additional conceptual frameworks and methodologies that identified vulnerability and potential hazards from systematic review and expert consultation which led to the creation of spatially explicit malaria risk maps were considered [9, 13–15]. In lieu of utilizing weighted indicators derived from the coefficients of regression analysis [13], the analytic hierarchy process (AHP) [16, 17] was used to obtain weights for the model’s 10 input parameters, or factors. AHP allows for weighing of attributes through pairwise comparisons of each factor against each other in order to rank the importance of each factor in multicriteria decision making [16–19] and was deemed an appropriate method of obtaining weights for the input factors selected to prioritize which LGAs should receive ITNs first.

A single cumulative prioritization map was produced after quantifying and weighting multiple *P. falciparum* infection risk factors, in lieu of individual malaria risk and hazard maps produced by Hanafi et al. [8] Additional risk factors pertaining to intervention coverage, socio-economic status, malaria prevalence, ITN access, built-up area presence index, and location of internally displaced populations (IDPs) were included in the creation of LGA prioritization maps. These factors were used to characterize LGAs according to their risk of malaria transmission based on their vulnerability for breeding and maintenance of malaria vectors, levels of intervention coverage, and social and biological susceptibility factors. Natural breaks, or Jenks, in the data were used to categorize values for each factor. Jenks natural breaks algorithms are commonly used in application of GIS data and optimize the arrangement of values into classes based on natural groupings of data values for a given variable [20]. This method has been adopted in multiple instances for classifying or categorizing geographic units based on distribution of data values [8, 12, 21, 22].

The steps below summarize the approach used to calculate final risk scores for each LGA. Step-by-step details of the implementation of the approach using R software are provided under a separate section (Calculation of prioritization scores).

- Most relevant risk factors that need to be considered for prioritization of planned vector control interventions (e.g., ITN campaign) were identified. The types and number of factors used for prioritization could differ between countries and interventions.
- The possible range of values of each factor was classified into a number of classes (typically four). For example, a factor such as ‘Seasonal Malaria Chemoprevention (SMC) implementation in 2019’ had two classes (‘not implemented’ and ‘implemented’), whereas ‘percentage of children aged 6–59 months who tested positive for malaria by microscopy’ could have five classes (e.g., > 42%, 33–42%, 23–32%, 13–22%, and < 12%). Where appropriate, cut-off values were determined using ‘natural breaks’ (where classes are based on natural groupings inherent in the data) as obtained using the ‘BAMMTools’ package [23] within R software (R Studio version 4.0.3).
- Rank values, defined here as whole numbers typically ranging from 1 to 4 (but could be 0 and 1 for a binary factor, denoting absence or presence of a particular intervention, for example), were assigned to each class (grouping of values based on Jenks) of each factor. The higher the rank value the greater the malaria risk associated with that class. For example, the rank value of the class ‘ > 42%’ for the factor percentage of children aged 6–59 months who tested positive for malaria by microscopy mentioned above would be 5, whereas ‘ < 12%’ would be assigned a rank value of 1.
- A short questionnaire was circulated among malaria professionals and academics during focus group interviews to ask them to rank each factor in terms of importance for quantifying malaria risk. These scores were translated into Saaty values and applied in an AHP analysis to obtain specific weights for each factor. Details of this process are described below in the section on obtaining weights for input factors.
- Each LGA was then assigned rank values for each factor, which was multiplied by the respective weight of the factor to obtain the risk scores. Risk scores of each factor were then summated to generate a composite prioritization score for each LGA.

Classification intervals featured in Table 1 were calculated using data from Nigeria as an example. This work aims to provide a generalized approach that can be adopted in countries and regions that may have access to various data sources or those that may adopt distinct malaria control activities. For example, SMC is typically conducted in limited regions, therefore this data input would not be relevant for countries or regions where SMC interventions are not implemented.

### Data inputs

Data related to each of the factors were obtained from various sources, including national malaria control strategic plans, ITN operational plans, vector control coverage data, and entomological and epidemiological reports. Information used from these documents featured geographic distribution of malaria interventions in Nigeria, such as distribution of piperonyl butoxide (PBO) nets in specific regions, number of years since the last mass ITN distribution campaign for each state, and SMC coverage. Literature relevant to models and approaches used to quantify and target ITNs in malaria-endemic settings were also reviewed in detail [8, 13–15]. Information from these data sources were used to characterize malaria risk and inform the approach utilized for developing the ITN distribution prioritization scheme in Nigeria.

A combination of DHS data [24], intervention coverage data, IDP data [7], environmental covariate data [6], and predicted surface layers [5, 25] were used as the primary data inputs to generate prioritization maps for Nigeria. A temperature suitability index for *P. falciparum* transmission was obtained from MAP [5]. Mean annual rainfall for each LGA was calculated using CHIRPS data [6]. Intervention coverage data was provided by Nigeria’s National Malaria Elimination Programme (NMEP). Built-up area presence data [25] from the Global Human Settlement Layer (GHSL) Project produces global spatial information about the human presence on the planet over time and relies on automatic analysis of satellite imagery to produce fine-scale maps quantifying built-up structures in terms of their location and density, and was used as a proxy for the classification of rural and urban areas.

Whether or not LGAs received PBO nets in a previous ITN distribution was used as a proxy for the presence of insecticide resistance. Other entomological data, such as density and distribution of *Anopheles* species, or species-specific behaviour, was not available for the entire country. In the case of Nigeria, inclusion of this entomological data would have required substantial modelling, such as spatial interpolation of data collected from entomological survey sites in order for it to be incorporated into the model. The data were, therefore, excluded from the algorithm, although it is recommended that the data be incorporated (if possible) if this exercise is conducted in other settings or countries.

An ITN access layer was interpolated using survey data [24] collected through the most recent Nigeria DHS conducted in 2018. Values obtained from this modelled layer were compared to state-level ITN access data as presented in the 2018 DHS [26] to identify alternative mechanisms for quantifying ITN access in the event that a spatially interpolated layer cannot be created. Malaria prevalence data for each state, defined as the percentage of children aged 6–59 months who tested positive for malaria with microscopy, was obtained from the most recent DHS [26]. Percentage of the *de jure* population within the lowest wealth quintile was used to represent socio-economic status and was also derived from state-level percentages reported in the latest (2018) DHS [26]. Distribution of IDPs per state was obtained from the Humanitarian Data Exchange [7].

### Obtaining weights for input factors

To quantify the degree that each factor should contribute to informing prioritization, focus group interviews were conducted among regional and global malaria experts. Respondents were asked to rank, on a scale of 1–10, the importance of each factor in terms of its relevance in contributing to malaria risk. The difference in the scores assigned to each factor by survey respondents were then translated into a Saaty value [19] through pairwise comparisons. For example, if the mean score for ITN access was 8, and the mean score implementation of SMC was 5, the difference in mean scores for these factors would be 3, with ITN access being deemed more important in characterizing malaria risk than implementation of SMC. A difference of 3 points would translate into a Saaty rating of 4 [19] (Table 2). In the event that a comparison variable was ranked higher, the reciprocal of the difference in scores was used. Table 2 illustrates how differences in scores obtained through pairwise comparisons were translated into Saaty values for use in the AHP.

**Table 2:** Scheme for translation of survey response scores into Saaty numerical ratings

Scale	Point difference in scores of pairwise comparison variables	Numerical rating (Saaty value)	Reciprocal
Extremely preferred	8	9	1/9
Very strong to extremely	7	8	1/8
Very strongly preferred	6	7	1/7
Strongly to very strongly	5	6	1/6
Strongly preferred	4	5	1/5
Moderately to strongly	3	4	1/4
Moderately preferred	2	3	1/3
Equally to moderately	1	2	1/2
Equally preferred	0	1	N/A

The ‘ahpsurvey’ package [27, 28] in R studio was used to calculate the aggregated preference weights of each factor, using a canned approach [28]. A component of the AHP involves normalization of the pairwise comparison matrix so that the summation of weights for factors equals 1 [19]. The contribution of each factor in characterizing malaria risk was determined through calculation of eigenvectors, which represents the relative weights between each factor [19] (Table 3). Weights obtained through calculation of eigenvectors using the ahpsurvey package were deemed appropriate as all observations were deemed consistent after meeting the pre-specified consistency ratio censoring threshold of 0.1 [28]. Final weights adopted by applying the AHP are presented in Tables 1 and 3.

**Table 3:** AHP aggregated priority weights for prioritization scheme input factors

Factor (input parameter)	Eigenvector (aggregated priority weight)	Weight (%)
Number of years since last ITN distribution	0.18	18.1
Rainfall	0.16	16.0
ITN access	0.15	14.7
PBO nets distributed	0.10	9.6
*P. falciparum* temperature suitability index	0.09	9.0
Built-up area index	0.09	8.7
SMC	0.08	8.1
*P. falciparum* prevalence rate	0.07	6.8
Mean wealth index	0.06	6.2
Presence of IDPs	0.03	2.7
Total	1.00	100.0

### Spatial interpolation

A major component that influenced the prioritization scheme was previous ITN access. This layer was obtained through spatial interpolation of 2018 DHS data on the proportion of the household population who could sleep under an ITN if each ITN in the household were used by up to two people [29]. Covariate raster layers used to create this interpolated layer are featured in Additional files 2, 3, 4 and 5. Using the R-INLA package, a Bayesian inference spatial smoothing approach called Integrated Nested Laplace Approximation with Stochastic Partial Differential Equation (INLA-SPDE) was used to fit a geostatistical model predicting the proportion of households with at least 1 ITN per 2 de facto household population at unsampled locations. This process was conducted using cluster-level input data [24] from the most recent DHS which collected information on household-level ITN access. DHS clusters were georeferenced and cluster-level estimates served as marked data points. DHS and MIS data generally allow for the calculation of representative estimates at the national, regional and urban/rural levels, but the INLA process allows for pixel-level estimates, which can be aggregated to establish sub-regional estimates based on available administrative boundaries. A logistic regression model was fit in INLA to estimate the probability of ITN access across the mesh while accounting for spatial autocorrelation. Also included in the INLA function was an estimation stack comprised of the cluster-level ITN estimates as well as corresponding covariate estimates. Raster values were extracted for each DHS or MIS cluster point using a 2-km buffer for urban clusters and a 10-km buffer for rural clusters. These specific buffer values were adopted to compensate for the geographic displacement of survey cluster point coordinates while extracting raster data, as recommended in the DHS Geospatial Covariate Datasets Manual [30]. The final output generated was a surface layer featuring predicted proportion of households with at least 1 ITN per 2 persons. This layer was combined with other factors, as listed in Table 1, to generate a final prioritization score for each LGA. The following section details how this was achieved.

### Calculation of prioritization scores

Table 4 presents a step-by-step framework illustrating the method for calculating prioritization scores in R Studio version 4.0.3, including only some of the important variables that could be used in most malaria-endemic countries (especially in Africa). Other country-specific factors could be included in the calculation alongside the input variables described below. Syntax for each step, including a list of required packages for calculation and application of prioritization scores in R Studio, can be found in the GitHub repository titled Tulane Malaria Consortium ITN prioritization methodology Nigeria [32] (see Additional file 1).

**Table 4:** Prioritization scheme framework featuring the list of steps required to create final prioritization maps

Step	Description
1	Load relevant R packages and libraries
2	Obtain administrative boundaries of country/region of interest by either loading a pre-existing shapefile or directly through GADM [33]
3	Obtain water boundary shapefile layers from OCHA Humanitarian Data Exchange [34] (if relevant)
4	Obtain temperature suitability index raster layer from Malaria Atlas Project [5, 35]. If a temperature suitability index raster is not available or accessible, a combination of mean monthly rainfall, land surface temperature, and elevation can be used
5	Obtain monthly mean rainfall raster layers from CHIRPS [6]
6	Import spatially interpolated raster layer featuring proportion of households with at least 1 ITN per 2 people (if available). This is created using cluster-level DHS or MIS data [24] pertaining to ITN use and raster layers of covariates [5, 35] such as travel time to nearest city of 60,000 or more inhabitants, 2015 educational attainment for women of reproductive age, 2015 prevalence of improved housing, and the 2015 settlement model (a combination of built environment and population density), via the INLA-SPDE package
7	Aggregate relevant indicators (see Table 1 for examples) to desired administrative boundary level and add to a master dataset that is linked to the name and unique code for the desired administrative boundary. The unit which data is aggregated to is dependent upon the resolution of intervention data available (for example number of persons receiving SMC per local government area). Ensure that desired administrative boundary shapefile exists prior to aggregating values to desired administrative level
8	Convert categorical variable to most appropriate categorical variables from literature review and/or use natural breaks from ‘getJenksBreaks’ function in R with desired number of classes
9	Assign rank values to indicators, keeping in mind that the complement (or inverse) of some indicators may need to be calculated in order to maintain consistency with increased or decreased risk scores. For example, increased rainfall values (larger positive value) may imply increased malaria risk while, high population density, high urbanization (larger positive value) may imply decreased malaria risk especially if the main vector’s preferred larval habitats are in rural settings
10	Obtain factor-specific weights through application of the AHP or Delphi method. See GitHub repository [31] for syntax on calculating AHP weights using the ‘ahpsurvey’ package in R. Multiply weight value (as featured in Table 1) by rank values for each factor to create factor-specific score
11	Summate final factor-specific risk scores for each administrative unit so that each administrative boundary has cumulative prioritization score where a higher total score is indicative of higher prioritization for ITN targeting

## Results

### Categories and weighting

Jenks natural breaks algorithms were used to categorize LGAs into one of four classes for the following factors: CHIRPS mean annual rainfall (mm) from May 2019 to April 2020, *P. falciparum* temperature suitability index, number of years since last mass ITN distribution, proportion of households with at least 1 ITN per 2 people, built-up area presence index, and percentage of *de jure* population comprising the lowest wealth quintile. Five intervals were used to classify which LGAs fell under each interval for percentage of children aged 6–59 months who tested positive for malaria by microscopy, as presented in the most recent DHS report for Nigeria. LGAs were dichotomously categorized for the following factors: presence of IDPs, distribution of PBO ITNs and implementation of SMC.

Results from focus group interviews and AHP analysis yielded aggregated factor weight values ranging from 0.03 to 0.18 (Tables 1, 3). Factors that were given the largest influence on the final prioritization scheme included number of years since last ITN distribution, mean annual rainfall, and proportion of households with at least 1 ITN per 2 people (aggregated AHP weights ranging from 0.15 to 0.18) (Tables 1, 3). The lowest weight (0.03) was applied to presence of IDPs. Application of the AHP to focus group survey responses returned a lower aggregated priority weight of 0.07 for a key malaria risk factor: percentage of children aged 6–59 months who tested positive for malaria by microscopy. Spatial distribution of LGAs by factors that contributed the most numerical value to each’s final prioritization score can be found in Additional file 6.

### Prioritization schemes and outputs

The prioritization score calculation framework was used to create classifications for each of the factors (input parameters) featured in Table 1 and identify LGAs that fell within each factor’s classification interval. Figure 1a–k illustrates the geospatial distribution of each of the factors and their corresponding classifications. There was minimal geographic correspondence between factors, excluding mean annual rainfall (Fig. 1a) and *P. falciparum* temperature suitability index (Fig. 1b). A majority of LGAs that had not received a mass ITN distribution in more than 6 years (Fig. 1c) also observed a higher density of IDPs (Fig. 1g).

Using the data inputs featured in Fig. 1a–j and Table 1, a final prioritization map was created (Fig. 2a) that illustrates the spatial distribution of all 775 LGAs in Nigeria and their characterization as being extremely high, high, ‘moderate-high’, ‘moderate’, ‘moderate-low’, or ‘low’ priority in terms of which should be targeted first in a mass ITN distribution in Nigeria. Some 15.5% of all LGAs (n = 120) were categorized as being extremely high or high priority. The majority of LGAs fell under the categorization of moderate priority (36.1%, n = 280), and less than 1% of LGAs were considered low priority (n = 6) (Table 5).

**Table 5:** Distribution of LGAs by priority class and ITN access layer

Prioritization category	ITN access obtained from spatial interpolation of 2018 DHS cluster data n (%)	ITN access obtained from state level percentages reported in 2018 DHS report n (%)
Extremely high	4 (0.5)	21 (2.7)
High	116 (15.0)	89 (11.5)
Moderate high	223 (28.8)	147 (19.0)
Moderate	280 (36.1)	337 (43.5)
Moderate low	146 (18.8)	180 (23.2)
Low	6 (0.8)	1 (0.1)
Total	775 (100.00)	775 (100.00)

### Sensitivity analysis using interpolated high-resolution *versus* reported low-resolution ITN access layers

To determine the added value of creating a spatially interpolated layer featuring estimated ITN access, a second prioritization map was created that featured the readily available state-level ITN access data as presented in the 2018 DHS Nigeria report. This second prioritization map was created using the same input factors as the original prioritization map, however ITN access was quantified in this instance at the state, as opposed to LGA level, using ITN access percentages featured in Nigeria’s 2018 DHS report. When using state level percentages to quantify ITN access, 25.2% of LGAs (n = 195) were classified in a discordant risk category when compared to outputs produced using a spatially interpolated ITN access layer. However, no LGA was categorized at a prioritization level of more than one interval higher or lower than their original ranking. When using DHS-reported state level ITN access as an input, 21 LGAs (2.7%) were categorized as extremely high priority in comparison to 4 (0.5%) when the interpolated ITN access layer was used. Some 11.5% of LGAs (n = 89) were categorized as high priority compared to 15.0% of LGAs (n = 116) when an interpolated layer was used. When using the spatially interpolated ITN access as an input, a greater number of LGAs were classified as low priority (6, 0.8% *vs* 1, 0.1%). However, when the highest and lowest prioritization classes were combined, minimal variance was observed in the distribution of LGAs per prioritization class, especially among the two classes with the highest priority. For example, 120 LGAs were categorized as extremely high or high priority when the interpolated ITN access layer was used. When scores were calculated using the DHS-reported state level ITN access layer, 108 (90.0%) of these 120 LGAs were also categorized as being extremely high or high priority. A similar, however inversed, trend was observed when low and moderate-low categories were combined. When compared to the spatially interpolated ITN access layer, the DHS-reported state level ITN access layer categorized more LGAs as being low or moderate-low priority, with a total of 181 LGAs falling within one of these categories. Of these 181 LGAs, 142 (78.0%) were also categorized as low or moderate-low when the spatially interpolated layer was used to quantify ITN access. The distribution of LGAs per risk category resulting from each layer can be found in Table 5. Visual representation of the differences in final LGA prioritization classifications using (a) the spatially interpolated layer *versus* (b) state-level reported percentages are shown in Fig. 2.

### Application of results

Figure 2 features spatial distribution of LGAs by prioritization class based on final prioritization scores. The majority of LGAs categorized as being high or extremely high priority were featured in the south of the country. It is recommended that LGAs with final prioritization scores that corresponded with risk classification of extremely high or high risk be prioritized for ITN distributions in the short term and other LGAs with lower priority levels be targeted in subsequent years.

## Discussion

The method presented during this exercise provides a practical approach to identify geographic areas that should be prioritized for mass ITN campaigns. Sensitivity analysis utilizing an alternative layer characterizing ITN access at a state level did not modify the outputs substantially, especially for LGAs characterized as extremely high and high priority. In this case study using data from Nigeria, increasing trends in mean annual rainfall were spatially consistent with increasing trends in *P. falciparum* temperature suitability index classification. Minimal spatial correlation was visually observed between presence of internally displaced populations and LGAs characterized as higher priority; however, the presence of internally displaced populations was the least weighted indicator. The most recent DHS survey indicated generally higher malaria prevalence rates in northern states compared to southern states, although climatic conditions in the latter are relatively more suitable for transmission. Heightened malaria prevalence observed in the north may be associated with other factors such as socio-economic status, expressed here as the distribution of the percentage of population comprising lowest wealth quintile, which is substantially higher in the northern part of the country than the south (Fig. 1i). Political violence, armed conflict, socio-economic vulnerability and disruptive events [32] in the northeast of the country may also explain the higher malaria prevalence rates observed in this region. Lower malaria prevalence in the southern region of the country may be influenced by increased population density and higher rates of urbanization in this region, despite the homogenous distribution of built-up area presence throughout the country (Fig. 1f).

Distribution of LGAs categorized as higher priority is limited primarily to the south of the country. This may have been driven largely by the fact that many LGAs where ITN campaigns have not been conducted for more than six years were located in this region. Additionally, time since last ITN distribution was among the highest weighted input factor. Another factor that may explain why southern LGAs were generally categorized as a higher priority is that SMC has only been implemented in the Sahel (northern) region of Nigeria. Spatial layers indicating increased risk due to mean annual rainfall, *P. falciparum* temperature suitability index, number of years since last ITN distribution, history of PBO net distribution in 2019, ITN access, and lack of SMC interventions aligned with expectations that some areas in the southern regions of the country would require increased prioritization for vector control interventions. Higher prioritization areas in the northeast region of Nigeria may have been largely influenced by the number of years since last ITN distribution, presence of internally displaced populations, and ITN access due to the cumulative coordination of spatial layers.

The spatially interpolated layer that features estimated ITN access was a major component in informing regions that should be prioritized in ITN distributions. This layer does, however, require a certain amount of modelling to create, and therefore differs from the other factors that were used to inform targeting. When priority classes were combined, LGAs categorized as extremely high or high priority and low or moderate-low priority did not vary substantially based on the layer used to quantify ITN access. The minimal variation in LGAs categorized as extremely high to high indicate that the use of aggregated DHS or MIS data on the proportion of households with at least 1 ITN per 2 people serves as a suitable alternative for quantifying ITN access in high-risk regions in the event that a spatially interpolated layer cannot be created. Furthermore, DHS-reported state level ITN access provided a more conservative estimate of ITN access for the highest prioritization class (extremely high priority). Due to the depreciation of ITN effectiveness over time, it is recommended that DHS or MIS survey data older than 3 years should be used with caution to quantify ITN access. If survey data older than 3 years are used, a function that accounts for net decay should be integrated.

Despite higher malaria prevalence reported in the north the outputs show that LGAs that were classified as higher priority were located primarily in the south. Furthermore, data inputs that more directly reflect malaria risk, such as malaria prevalence could be assigned higher weight values, although AHP weighting results concluded a lower priority for this input layer. Lower weight for this prevalence indicator could be justified due to the malaria prevalence indicator’s limitations in that the 2018 DHS was implemented over approximately 4.5 months, extending into the dry season in some areas, therefore may not accurately reflect true malaria prevalence in all areas.

Whereas this approach was initially designed for targeting which areas should receive ITNs first (extremely high and high priority) in upcoming mass distributions amidst a public health emergency, its scope could be adapted for targeting of a wider range of vector control interventions to optimize the use of limited resources, especially in regions experiencing increased insecticide resistance and the need to use expensive tools and strategies. The strategy presented here could be adapted and applied in a variety of public health emergency and disease outbreak settings.

## Limitations

Data on distribution of community health workers (CHWs) offering malaria test-and-treat services was not available and was therefore not included as an input in calculation of final prioritization scores. Indicators, such as confirmed malaria cases, were not included in the analysis for generating prioritization maps as malaria morbidity data collected through routine health facility-based malaria surveillance were believed to not accurately represent case burden due to incomplete reporting. In the case of Nigeria, entomological data such as density and distribution of *Anopheles* species were only available for a number of entomological survey sites. Inclusion of specific entomological data in the model would have required additional modelling and spatial interpolation, therefore these inputs were minimized in order to expedite LLIN prioritization outputs during a public health emergency. Consequently, focus was placed on environmental covariates, DHS data and malaria intervention coverage to assess malaria risk. Given the necessity to rapidly produce outputs to inform ITN distribution during the commencement of the COVD-19 pandemic, in-depth evaluation of the accuracy of prediction of malaria risk using environmental factors, as well as the impact of inclusion or exclusion of some factors on the prioritization index was not conducted.

## Conclusions

This paper presents a decision-making tool that can be used to prioritize regions for ITN distribution, especially during public health emergencies and in resource-limited settings. Whereas there are some limitations in terms of the factors included to prioritize regions for ITN distribution in Nigeria, the authors believe that they have proposed a valuable and practical tool for ITN targeting as it can be used to produce outputs in a limited time frame, while using a range of indicators that are typically available and systematically collected in countries that experience *P. falciparum* transmission. Results from this exercise suggest that factors outside of those that exclusively characterize risk of malaria transmission and maintenance of malaria vectors, for example, concurrent intervention coverage and presence of disruptive events, should also be considered when targeting ITN distributions. Not only does this research present a framework that can be adapted to variety of country-contexts, but it also provides a baseline methodology that could be adjusted for targeting a wider range vector control interventions.

## Supplementary Information

**Additional file 1.** GitHub repository: Tulane Malaria Consortium ITN prioritization methodology Nigeria. Syntax and code for each step listed in the prioritization framework (Table 4) can be found in this public GitHub repository titled Tulane Malaria Consortium ITN prioritization methodology Nigeria

**Additional file 2.** 2015 Settlement Model. 2015 Settlement Model (SMOD) covariate raster layer utilised in creation of spatially interpolated ITN access layer

**Additional file 3.** Prevalence of Improved Housing. 2015 Prevalence of Improved Housing covariate raster layer utilised in creation of spatially interpolated ITN access layer

**Additional file 4.** Travel time to nearest city of 60,000 or more inhabitants. Travel time to nearest city of 60,000 or more inhabitants covariate raster layer utilised in creation of spatially interpolated ITN access layer

**Additional file 5.** 2015 mean years of educational attainment for women aged 15–29 years. 2015 mean years of educational attainment for women aged 15–29 years covariate raster covariate raster layer utilised in creation of spatially interpolated ITN access layer

**Additional file 6.** Most influential factors contributing to unique LGA prioritization scores. Chloropleth map featuring the spatial distribution of the most influential input factor during calculation of the final LGA prioritization score. This was obtained by calculating the proportion each input factor value contributed to the final prioritization score. ITN dist corresponds with number of years since last mass ITN distribution, ITN access represents the proportion households with at least 1 ITN per 2 people (interpolated layer), TSI corresponds with Plasmodium falciparum suitability index, and SMOD corresponds with built-up area presence (used as a proxy for rural/urban designation). If a combination of factors is listed as the most influential factor, then these factors contributed the same amount to the final prioritization score.

## Data Availability

The datasets used and/or analysed during the current study are available from the corresponding author on reasonable request.

## Abbreviations

AHP: Analytic hierarchy process
CHIRPS: Climate Hazards Group InfraRed Precipitation with Station
CHW: Community health worker
DHS: Demographic Health Survey
GHSL: Global Human Settlement Layer
GHSL-SMOD: Global Human Settlement Layer Model Grid
GIS: Geographic information system
IDP: Internally displaced population
INLA-SPDE: Integrated Nested Laplace Approximation with Stochastic Partial Differential Equation
IRS: Indoor residual spraying
ITN: Insecticide-treated net
LGA: Local government area
LST: Land surface temperature
MAP: Malaria Atlas Project
MIS: Malaria Indicator Survey
MOH: Ministry of Health
NMCP: National Malaria Control Programme
NMEP: National Malaria Elimination Programme
PBO: Piperonyl butoxide
SMC: Seasonal malaria chemoprophylaxis
