# Supplementary material for: A practical approach for geographic prioritization and targeting of insecticide-treated net distribution campaigns during public health emergencies and in resource-limited settings
Source: Malar J. 2022 Jan 4;21:10. doi: 10.1186/s12936-021-04028-y (PMC8724754; doi:10.1186/s12936-021-04028-y)
Supplement: Supplementary file 1 — Additional file 1. GitHub repository: Tulane Malaria Consortium ITN prioritization methodology Nigeria. Syntax and code for each step listed in the prioritization framework (Table 4) can be found in this public GitHub repository titled Tulane Malaria Consortium ITN prioritization methodology Nigeria [file 12936_2021_4028_MOESM1_ESM.docx]

### Additional file 1

Syntax and code for each step listed in the prioritization framework (Table 2) can be found in the public GitHub repository titled “Tulane Malaria Consortium ITN prioritization methodology Nigeria”, and can be accessed using the following link: <https://github.com/alyssajyoung789/Tulane_MalariaConsortium_ITN_prioritization_methodology_Nigeria>
