# Supplementary figures and images for: A practical approach for geographic prioritization and targeting of insecticide-treated net distribution campaigns during public health emergencies and in resource-limited settings
Source: Malar J. 2022 Jan 4;21:10. doi: 10.1186/s12936-021-04028-y (PMC8724754; doi:10.1186/s12936-021-04028-y)

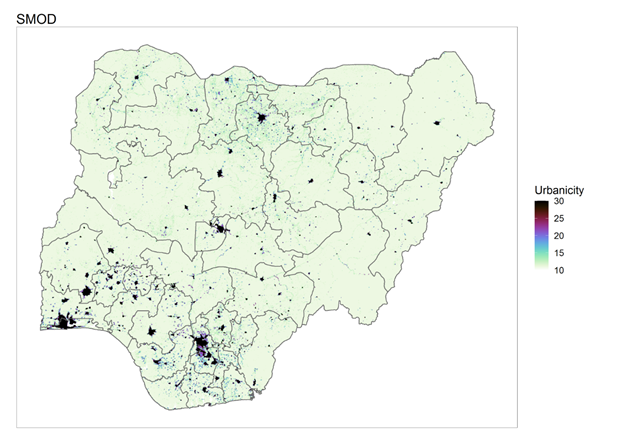

Supplement: Supplementary file 2 — Additional file 2. 2015 Settlement Model. 2015 Settlement Model (SMOD) covariate raster layer utilised in creation of spatially interpolated ITN access layer [file 12936_2021_4028_MOESM2_ESM.png]

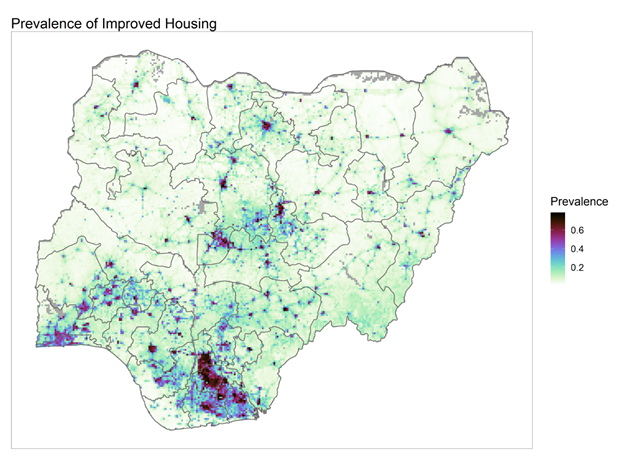

Supplement: Supplementary file 3 — Additional file 3. Prevalence of Improved Housing. 2015 Prevalence of Improved Housing covariate raster layer utilised in creation of spatially interpolated ITN access layer [file 12936_2021_4028_MOESM3_ESM.png]

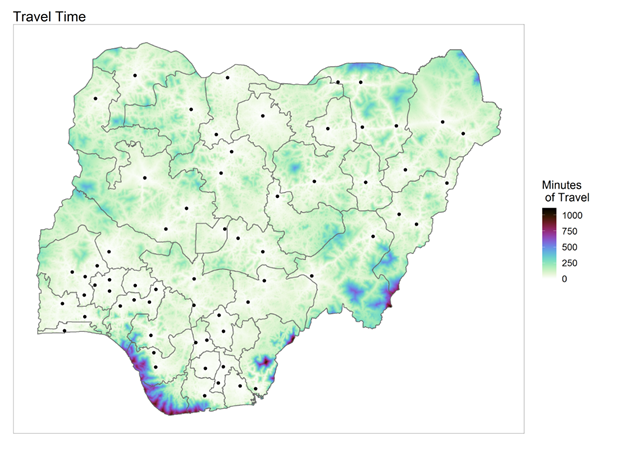

Supplement: Supplementary file 4 — Additional file 4. Travel time to nearest city of 60,000 or more inhabitants. Travel time to nearest city of 60,000 or more inhabitants covariate raster layer utilised in creation of spatially interpolated ITN access layer [file 12936_2021_4028_MOESM4_ESM.png]

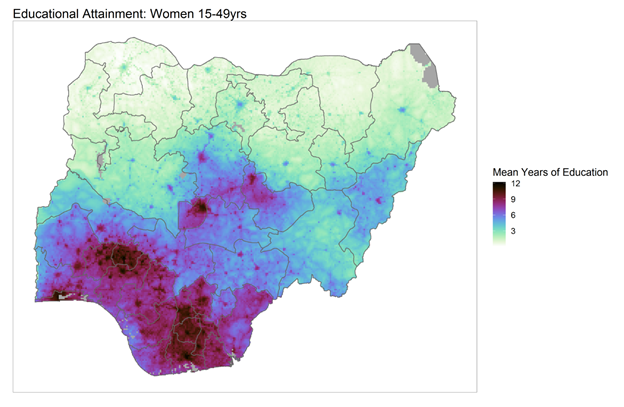

Supplement: Supplementary file 5 — Additional file 5. 2015 mean years of educational attainment for women aged 15–29 years. 2015 mean years of educational attainment for women aged 15–29 years covariate raster covariate raster layer utilised in creation of spatially interpolated ITN access layer [file 12936_2021_4028_MOESM5_ESM.png]

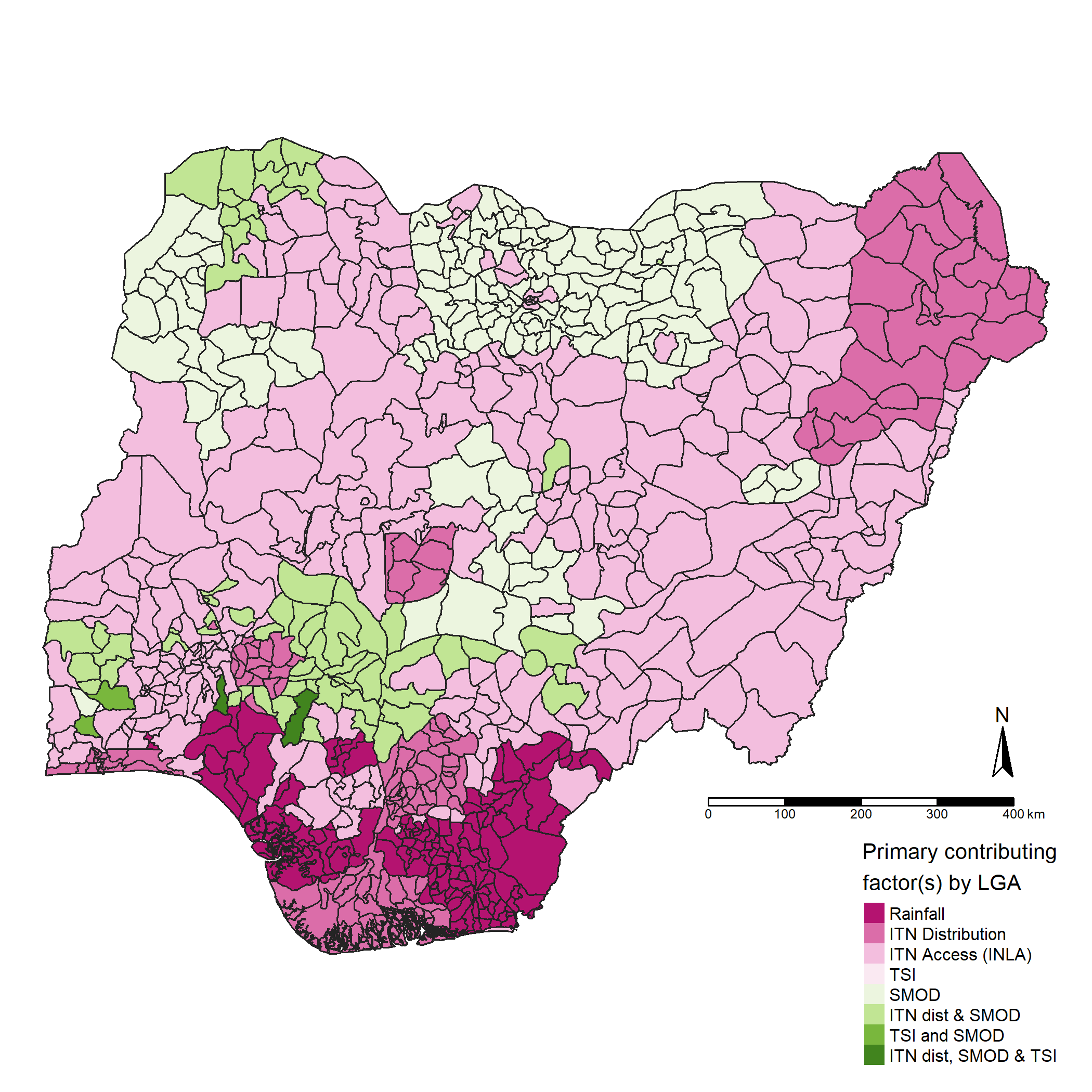

Supplement: Supplementary file 6 — Additional file 6. Most influential factors contributing to unique LGA prioritization scores. Chloropleth map featuring the spatial distribution of the most influential input factor during calculation of the final LGA prioritization score. This was obtained by calculating the proportion each input factor value contributed to the final prioritization score. ITN dist corresponds with number of years since last mass ITN distribution, ITN access represents the proportion households with at least 1 ITN per 2 people (interpolated layer), TSI corresponds with Plasmodium falciparum suitability index, and SMOD corresponds with built-up area presence (used as a proxy for rural/urban designation). If a combination of factors is listed as the most influential factor, then these factors contributed the same amount to the final prioritization score. [file 12936_2021_4028_MOESM6_ESM.png]
